# CSF immune cell phenotypes and their related pathway activation differ by Alzheimers disease stage compared to normal controls

**DOI:** 10.1002/alz.092469

**Published:** 2025-01-03

**Authors:** Gurkan Bebek, Ziyin Zhao, Mi‐Hyun Hwang, Cornelia Bergmann, Jagan A. Pillai

**Affiliations:** ^1^ Case Western Reserve University, Cleveland, OH USA; ^2^ Cleveland Clinic, Cleveland, OH USA; ^3^ Cleveland Clinic Lou Ruvo Center for Brain Health, Cleveland, OH USA

## Abstract

**Background:**

There is significant interest in understanding the nature of the inflammatory response and its role in Alzheimers disease (AD) pathophysiology. Immune cell phenotypes and their key pathway activation by AD stage is unclear. We therefore evaluated immune cell phenotypes in the cerebrospinal fluid (CSF) and their transcriptional profile comparing AD‐dementia, Mild Cognitive Impairment (MCI)‐AD and normal cognition controls using transcriptomics.

**Method:**

We characterized the gene expression profiles of immune cells in the CSF using both bulk RNA sequencing (RNAseq) and complementary single cell transcriptomics (scRNAseq). First, in a cross‐sectional cohort study, including 6 individuals with MCI‐AD meeting NIA/AA 2011 criteria and 9 amyloid negative controls with normal cognition, (mean age 68.75 years (SD 5.2), 58.8% Female), we identified significant pathways using gene set enrichment analysis (GSEA). Next, using scRNAseq analysis on2 AD‐dementia, 2 MCI‐AD and 2 normal controls we further elucidated the gene expression level changes in distinct immune cells. After clustering into immune cell types, we assessed their activation by GSEA and Gene set variation analysis (GSVA).

**Result:**

RNAseq analysis identified 47 genes that were differentially expressed between MCI‐AD and normal control samples (adj‐p‐val<0.05). GSEA identified downregulation of antigen processing, interferon signaling pathways, and neutrophil degranulation (adj‐p‐val<0.05). Next, scRNAseq, analysis defined immune cells within each sample and identified altered pathways that were in each cell type. We observed that CD4+CD8+ T cells were highly represented in AD‐dementia (17%) and MCI‐AD(14%) but not in normal controls(11%) (p<0.00001). The immune cell types that differed by disease stage between AD‐dementia and MCI‐AD were monocytes; increasing disease severity was associated with a transition from more classical monocytes to intermediate and nonclassical monocytes. The key pathways associated with monocytes were IL6 JAK STAT3 signaling, particularly STAT3, which is involved in monocyte‐to‐macrophage differentiation and the transition of classic monocytes to other subsets. Additionally, the pathways include interferon gamma (IFNg) response and TNF signaling via NF‐kB, which play important roles in activating macrophages and monocytes and promoting differentiation.

**Conclusion:**

Our data show enrichment in the proportion of CD4+CD8+ T cells and pathways related to monocyte differentiation that differ across AD stage.

